# Optical Coherence Tomography Characteristics Between Idiopathic Epiretinal Membranes and Secondary Epiretinal Membranes due to Peripheral Retinal Hole

**DOI:** 10.1155/joph/9299651

**Published:** 2025-05-07

**Authors:** Yuanyuan Fan, Yingying Jiang, Zhaoxia Mu, Yulian Xu, Ping Xie, Qinghuai Liu, Lijun Pu, Zizhong Hu

**Affiliations:** ^1^Department of Ophthalmology, The First Affiliated Hospital of Nanjing Medical University, Nanjing, Jiangsu 210029, China; ^2^Department of Ophthalmology, Zhangjiagang Hospital Affiliated to Soochow University, Suzhou, Jiangsu 215600, China

**Keywords:** epiretinal membrane, idiopathic, OCT, peripheral retinal hole, secondary

## Abstract

**Purpose:** In clinical practice, some eyes preoperatively diagnosed with “idiopathic epiretinal membranes (iERM)” will be amended to “secondary epiretinal membranes (sERM)” once peripheral retinal hole is detected. This study utilized optical coherence tomography (OCT) images to compare the characteristics between the iERM and sERM due to peripheral retinal hole (PRH).

**Methods:** In this retrospective, cross-sectional study, 635 eyes that had undergone pars plana vitrectomy with membrane peeling were enrolled. A total of 115 eyes (18.1%) detected with peripheral retinal holes were allocated to the sERM-PRH group while the other 520 eyes were to the iERM group. The demographic data and OCT characteristics were compared between the two groups. Besides, all the eyes were evaluated by a double-grading scheme: severity grading of ERM progression into four stages plus anatomical classification into three kinds of part-thickness macular holes associated with ERMs.

**Results:** No significant difference was found in age, gender, symptom duration, axial length, or best-corrected visual acuity between the two groups. There was also no difference concerning the features based on OCT, ranging from central macular thickness, the ratios of the photoreceptor inner/outer segment junction line defect, intraretinal fluid, cotton ball sign, to epiretinal proliferation. However, the native difference in parafoveal thickness between the temporal and nasal quadrants was observed in the iERM group, yet disappeared in the sERM-PRH group. Moreover, eyes between the two groups were distributionally similar in both grading scales.

**Conclusion:** Our results demonstrated that even OCT images could hardly provide effective clues for early differentiating sERM from iERM, which highlighted the necessity of a thorough pre- and intro-operative fundus examination of the peripheral retina for clinicians.

## 1. Introduction

Epiretinal membrane (ERM) is a common retinal condition characterized by semitranslucent and fibrocellular tissue excessively growing on the surface of the internal limiting membrane (ILM), frequently accompanied by retinal wrinkling [1, 2]. Updated by a meta-analysis of 13 population-based studies in 2017, the overall prevalence of ERM has reached 9.1% [3]. Serious vision loss such as reduced visual acuity (VA), blurred vision, distortion, and metamorphopsia can be caused once ERM progresses to affect the macular and perimacular region [4, 5]. Therefore, there is substantial attention garnered from the diagnosis and treatment of ERM.

ERM can be either idiopathic or secondary. The idiopathic ERM (iERM), sometimes called primary ERM, often occurs without any other ocular diseases, or pursuant to a posterior vitreous detachment (PVD) only [6]. Secondary ERM (sERM) is the result of an already existing ocular disorder, including previous intraocular surgery, retinal vascular disease, intraocular inflammation, retinal tears or detachment, ocular trauma, intraocular tumors, and others [2, 4]. sERM and iERM share different etiology, symptoms, surgical choices, and outcomes, with sERM associated with worse VA, younger age incidence, and higher postsurgical recurrence [7]. Thus, instant surgical intervention is a necessity and ERM/ILM double peel is more favorable for sERM [8]. However, sERM due to peripheral retinal holes is easily mistaken for iERM as the precise fundus examination of the peripheral retina after mydriasis may be neglected. Neglecting peripheral retinal holes may eventually delay the treatment and even lead to serious adverse events like retinal detachment [9].

Optical coherence tomography (OCT), as a noninvasive, rapid, high-resolution imaging modality, has revolutionized the definition, description, classification, diagnosis, and prognosis of ERM [10]. It has an edge over traditional subjective observation through slim-lamp microscopy for high sensitivity, detailed anatomical structures, and accurate quantitation [11, 12] and may help grab morphologic biomarkers of sERM due to peripheral retinal holes. However, to the best of our knowledge, there have been few studies comparing the characteristics between iERM and sERM due to peripheral retinal holes based on OCT images. Therefore, we designed this study aiming to identify the morphological differences of macular images between them, which may give a hint for digging out diverse modalities for early and timely diagnosis and treatment for sERM due to peripheral retinal holes.

## 2. Methods

This retrospective cross-sectional study was conducted at the department of ophthalmology of the First Affiliated Hospital of Nanjing Medical University. Ethics approval was obtained from the Ethics Committee of the Faculty of Medicine, Nanjing Medical University (2023-SR-086). The written informed consent was waived as the study is based on retrospective data.

### 2.1. Study Design and Patient Inclusion

A retrospective review of medical records was conducted for patients who were diagnosed as ERMs and had undergone pars plana vitrectomy with membrane peeling between February 2015 and November 2022 at the First Affiliated Hospital of Nanjing Medical University. ERM was diagnosed clinically by slit-lamp fundus examination with a 90-diopter lens and OCT. The patients were classified into two groups: the iERM group and the sERM due to peripheral retinal hole (sERM-PRH) group. The iERM group included those who were diagnosed as ERM without a history of trauma, retinal detachment surgery, uveitis, diabetic retinopathy, retinal vein occlusion, or other underlying maculopathy. The sERM-PRH group enrolled those sERM patients who were detected with retinal hole in the clinic or those who were detected intraoperatively and received laser photocoagulation during the surgery. All the records were collected from their medical charts. The exclusion criteria were subjects with (1) concurrent diagnoses of diabetic retinopathy, retinal detachment, trauma, glaucoma, or full-thickness macular hole, (2) history of cataract surgery, and (3) an incapability of keeping eyeballs stable, undergoing ocular examinations, or producing good-quality OCT images.

### 2.2. Clinical Data

The basic clinical data of each subject were carefully collected from their medical charts, such as age, gender, systemic disease, duration of symptom, and laser photocoagulation for retinal holes. A comprehensive ophthalmic evaluation was performed, including best corrected visual acuity (BCVA), a refraction test using autorefractometer RC-5000 (Tomey, Nagoya, Japan), and phoropter RT-5100 (Nidek, Tokyo, Japan), AL measurement using Nidek-AL Scan optical biometry (Nidek, Gamagori, Japan), anterior segment examination using the Haag–Streit BM 900 slit-lamp microscope (Haag-Streit, Köniz, Switzerland), dilated fundus examination, and OCT (Optovue Avanti, Fremont, California, USA & Carl Zeiss Meditec, Dublin, California, USA). VA was converted into logarithm of the minimum angle of resolution (logMAR) for performing the statistical analysis. VA of light perception (LP) was assigned as 2.9 logMAR, hand movements (HMs) as 2.6 logMAR, and counting fingers (CFs) as 2.3 logMAR [13].

### 2.3. CMT Thickness

OCT images were acquired in all participating patients. Automated analyzing software embedded in each device was used to identify and delineate separate retinal layers, including ERM, ILM, and retinal pigment epithelium (RPE) layers. The central macular thickness (CMT) was also automatically calculated as the distance from inner surface of the retina to inner border of RPE within 1 mm centered to the fovea and the parafovea thickness was measured within 1–3 mm centered to the fovea.

### 2.4. OCT Characteristics

**Figure 1:**
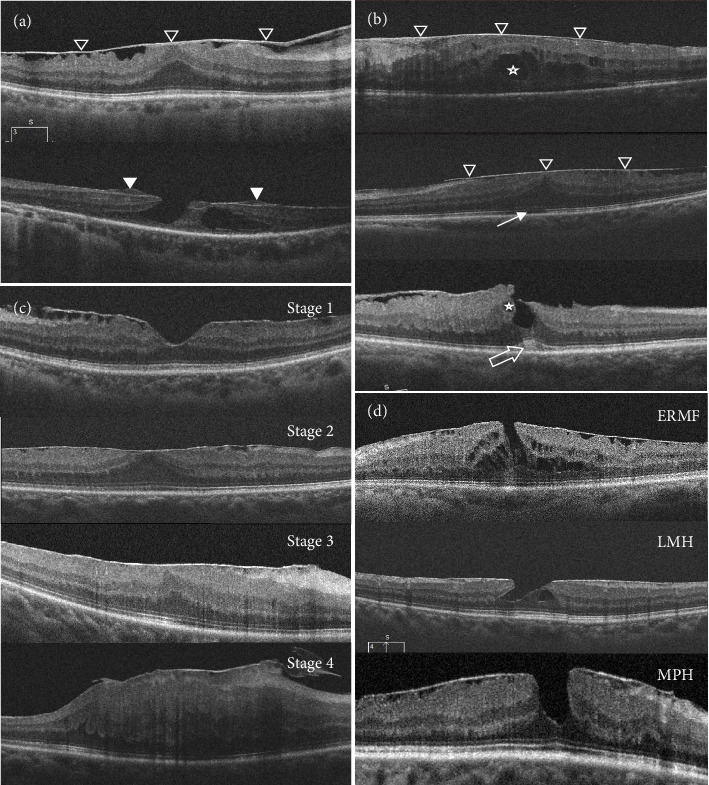
Signs on OCT. (a) Traditional ERM (black triangle) and epiretinal proliferation (white triangle). (b) IS/OS junction defect (white arrow), intraretinal fluid (star), and cotton ball sign (white hollow arrow). (c) Classification of ERM according to Govetto et al. [14]. (d) Classification of part-thickness macular hole according to Hubschman et al. [15].

Other parameters based on OCT, such as loss of the photoreceptor inner/outer segment (IS/OS) junction line, intraretinal fluid, cotton ball sign, type of ERMs, were also recorded. Traditional ERMs are fibroblastic cell proliferations on the inner surface of the macula which are characterized by increasing reflectivity on the surface of the retina, usually with signs of the underlying retina wrinkling (Figure 1(a)) [11]. EP, compared with normal ERM, has an unusual appearance and is characterized by a thick homogenous layer of moderately reflective material over the internal limiting membrane and often covered by a thin hyperreflective line [16] (Figure 1(a)). The intraretinal fluid was described as hyporeflective intraretinal cystoid spaces within retinal layers (Figure 1(b)). The IS/OS junction defect was defined as a discontinuous band of the photoreceptor inner/outer segment in the foveal region, which was considered closely related to poor visual function [17] (Figure 1(b)). The cotton ball sign was a roundish or diffuse highly reflective region between IS/OS junction line and cone outer segment tip line at the center of the fovea [17] (Figure 1(b)).

### 2.5. Double Grading Schemes for ERMs

On the one hand, we graded the clinical severity of all the OCT images from enrolled participant using the four-stage grading scheme proposed by Govetto et al. [14] in 2017. VA decreased in an inverse linear fashion as the ERM stage increased [18]. It is categorized based on the absence of a foveal pit, the existence of ectopic inner foveal layers (EIFLs), and the incoherence of the retinal layers (Figure 1(c)). Stage 1: there is a pit in the central foveal region and the retinal layers can be clearly defined. Stage 2: there are distinct layers of the retina but no foveal pit. Stage 3: the retinal layers are still clearly identifiable, the foveal pit is missing and a continuous EIFL has been added. Stage 4: the foveal pit is absent, an EIFL is present, and the retinal layers are disrupted. To note, EIFL is defined as the presence of a continuous hypo or hyper-reflective band extending from the inner nuclear layer (INL) and inner plexiform layer (IPL) across the fovea [14]. It is hypothesized that the EIFL develops following prolonged tractional stresses from the ERM, resulting in gliosis, Muller cell growth, and retinal architecture damage [19] and may be a novel indicator for metamorphopsia [20].

On the other hand, we found that many individuals in both the iERM group and sERM-PRH group showed part-thickness macular hole with ERM. We further turned for the anatomical grading scheme presented by Hubschman et al. [15] and classified them into three circumstances: epiretinal membrane foveoschisis (ERMF), lamellar macular hole (LMH), and macular pseudohole (MPH) (Figure 1(d)). ERMF was characterized by a sharp separation between the outer nuclear and outer plexiform layers, at the level of Henle's fiber layer, sometimes along with retinal wrinkling and microcystoid spaces in the INL, and the retina usually thicken. LMH was characterized by an irregular foveal contour and a foveal cavity with undermined edges along with apparent loss of foveal tissue, sometimes accompany with the presence of a central foveal bump and the interruption in the ellipsoid zone. MPH was characterized by a steepened foveal profile and an increased retinal thickness with a near normal central fovea thickness, sometimes with microcystoid spaces in the INL.

All the images were analyzed by one observer (YY. J.) blinded to patient information.

### 2.6. Statistical Analysis

Statistical analysis was carried out using IBM SPSS Statistics Version 25.0 (SPSS, IBM Software Group, Chicago, IL, USA). For all continuous variables, mean and standard deviation (SD) values were calculated and independent *t* test were used to compare the difference in age, AL, duration of symptom, BCVA, and CMT between the groups. Paired samples test was used to compare the difference of parafoveal thickness in opposite quadrants. Frequency and percentage were calculated for categorical variables. Pearson Chi-square test and continuity correction test were used to compare the differences. *p* value < 0.05 was considered statistically significant.

## 3. Results

### 3.1. Demographic Characteristics

A total of 635 eyes which had undergone pars plana vitrectomy with membrane peeling were enrolled in this study, with 520 allocated to iERM group (81.9%) and 115 to the sERM-PRH group (18.1%). As is illustrated in Table 1, there were no significant differences between two groups in terms of age, gender, eyes, duration of symptom, and combination with systemic diseases (all *p* > 0.05). The preoperative BCVA and AL were 0.84 ± 0.44 (logMAR) and 23.83 ± 1.76 mm in the iERM group and 0.93 ± 0.59 (logMAR) and 24.21 ± 2.17 mm in the sERM-PRH group, respectively. Generally, subjects in the sERM-PRH group seemed more myopic, yet no significant difference was observed (both *p* > 0.05).

### 3.2. Comparison of CMT in iERM and sERM-PRH Groups

The CMT of the iERM group were marginally higher than the sERM-PRH group (445.79 ± 114.61 μm for the iERM group and 435.13 ± 113.48 μm for the sERM-PRH group, *p*=0.366). The parafoveal thickness showed similar trend but the difference was also not statistically significant (Table 2).

In healthy population, the parafoveal thickness of temporal quadrant is usually thicker than that of nasal quadrant [21]. The difference of thickness may disappear as eye disease develops, which may make a hint for us to differentiate sERM-PRH from iERM. Our findings showed that the parafoveal thickness of temporal quadrant was higher than that of nasal quadrant in the iERM group (*p* < 0.001). On the contrary, this characteristic disappeared in sERM-PRH group (*p*=0.214) (Table 3).

### 3.3. Comparison of OCT Characteristics in iERM and sERM

As for the characteristics based on OCT images, there was no statistical difference between the two groups in the presence of IS/OS junction defect (*p*=0.518), cotton ball sign (*p*=0.419), intraretinal fluid (*p*=0.280), and epiretinal proliferation (*p*=0.972). Moreover, eyes between the two groups were distributionally similar in both grading scales (Table 4).

## 4. Discussion

In this single-center, retrospective study, we identified 18.1% ERMs as the sERM-PRH from 635 eyes which underwent vitrectomy with membrane peeling at our hospital. Binocular indirect ophthalmoscope in combination with the handled magnifying 90D lens is insufficient to see the entire retina, particularly pathological alterations adjacent to the ora serrata. Clinical observations indicate that peripheral retinal holes exhibit significantly reduced detection rates compared to central lesions (detection ratio: 1:2.048) [22]. These findings underscore the necessity for ophthalmologists to implement meticulous examination protocols during fundoscopic evaluations.

Retinal holes are roughly divided into two distinct categories: holes associated with PVD (horseshoe holes, operculated holes, and peripheral lamellar impairments) and atrophic holes (nonoperculated holes formed due to retinal thinning like lattice degeneration) [23]. For holes associated with PVD, Choudhry reported the partial vitreous adhesion at hole edges or opercula [24] and the axial traction from vitreous is likely to induce rhegmatogenous retinal detachment (RRD). Peripheral atrophic holes share a resemble appearance with idiopathic macular holes whose edges prune to be flat or everted, which probably explain their innocuous nature [25]. However, Govetto et al. [26] suggested atrophic holes linked to peripheral vitreoschisis were actually caused by tangential traction, also indicating high risk for the development of RRD. Therefore, no matter what kinds of retinal holes, they pose a threat to retinal detachment if the fluid in the vitreous cavity leaking into the subretina space [27]. Precisely locating the retinal holes and prompting laser photocoagulation is of crucial importance for treating sERM-PRH. Our retrospective cohort study predominantly identified chronic atrophic round or oval holes instead of newly developed ones, ruling out those combined with RRD, and performed laser photocoagulation during surgery. However, we failed to identify significant demographic, visual, or OCT characteristics differences between the iERM group and the sERM-PRH group, further indicating the necessity of careful peripheral examination for all ERMs.

sERM can occur secondary to retinal holes and other retinopathy or surgery. Previously, Yazici et al. [28] reported that the rate of sERM secondary to any ocular pathology was 57.34% in all ERMs (168 of 293 eyes), thus higher than our report. Another study showed the rate of sERM due to retinal holes was 36.56% (34 of 93 eyes with ERMs) [29]. Though this study split sERM due to retinal holes or breaks from sERMs like us, their results were also higher than that in our study. For one thing, we conducted a comprehensive retrospection with a larger sample size than theirs. For another thing, the patients in their studies were excluded if with cataracts of grade II or higher (Emery–Little classification). The rate in Dong Ik Kim's study was 19.19% (38 of 198 eyes with ERM) [30], similar with our result. Previous studies have reported that patients with the sERM were younger, with poorer VA and the CMT was also thicker than patients with iERM [28, 29]. Our findings, however, detected no difference in functional and morphological analysis between iERM and sERM-PRH, possibly due to the differences in population background, inclusion criteria, and sample size.

Few studies focused on the characteristic differences based on OCT between iERM and sERM-PRH. In the current study, most of the features based on OCT showed no difference between the two groups except that the native difference of the parafoveal thickness between the nasal and temporal quadrants disappeared in the sERM-PRH group. This may be caused by the uneven distribution of RPE cells and the eccentrically formed sERM [29], which exerts asymmetric traction forces to the retina. The eccentricity has also been demonstrated by en-face OCT and fundus photography in the study of Lee et al. [29] and Kim et al. [30]. Nevertheless, the abovementioned two studies did not further analyze the pattern of eccentric distribution of sERM. Future study is needed to further evaluate the site of ERM and its traction force to the retina.

Though the difference was hardly to distinguish in clinical practice, there might be a possibility that the artificial intelligence (AI) algorism can be developed based on mass OCT data to identify sERM-PRH and iERM. Recently, AI was able to identify different retinal lesions based on OCT images, such as cystoid macular edema, ERM, serous macular detachment, and macular hole [31]. In addition, the application of deep learning has the potential to further improve visual prognosis and pinpoint patients who would most benefit from surgery [32, 33]. But there are still no successful reports about AI differentiating sERM-PRH and iERM grounding on existing ophthalmic modalities, which underscored the pivotal role of extensive dilated fundus examination with a 90 D lens.

We had to admit that there were several limitations in this retrospective cross-sectional study. First, this was a single-center study only including ERMs after vitrectomy. Second, the postoperative prognosis was not followed up. Third, examinations such as en-face OCT, OCTA, and fundus fluorescence angiography were not implemented in our study. Fourth, the sample size of sERM was far smaller than that of iERM.

In summary, this study investigated characteristics based on OCT between iERM and sERM due to peripheral retinal holes with the aim to identify sERM from iERM. However, few differences were found in this study. This means that for clinicians, it is hard to identify sERM from iERM based solely on demographic and morphological data. Also, surgeons should keep in mind that there is an abundance of ERMs secondary to peripheral retinal holes, which calls for more detailed fundus examinations during surgery. In the future, consensus on the diagnosis for these retinal pathologies will be evolving and there will be decreasing ambiguity as newer technology becomes available.

## Figures and Tables

**Table 1:** Demographic and clinical characteristics.

	iERM (*N* = 520)	sERM-PRH (*N* = 115)	*p* value
Age (years)	64.83 ± 8.83	63.55 ± 10.16	0.172^a^
Females	346 (66.5)	74 (64.3)	0.653^b^
Duration of symptom (mons)	20.01 ± 26.89	19.16 ± 28.14	0.763^a^
OD	268 (51.5)	59 (51.3)	0.964^b^
BCVA (Log MAR)	0.84 ± 0.44	0.93 ± 0.59	0.136^a^
AL (mm)	23.83 ± 1.76	24.21 ± 2.17	0.092^a^

*Systematic diseases*			
Hypertension	148 (28.5)	37 (32.2)	0.428^b^
Diabetes	50 (9.6)	8 (7.0)	0.370^b^

*Note:* iERM = idiopathic epiretinal membrane; sERM-PRH = secondary epiretinal membrane due to peripheral retinal hole; LogMAR = logarithm of the minimum angle of resolution.

Abbreviations: AL, axial length; BCVA, best corrected visual acuity; OD, Oculus Dexter.

^a^Independent *t* test.

^b^Pearson Chi-square test.

**Table 2:** Central macular and parafoveal thickness.

	iERM (*N* = 520)	sERM-PRH (*N* = 115)	*p* value
CMT (μm)	445.79 ± 114.61	435.13 ± 113.48	0.366
Temporal (μm)	430.03 ± 96.10	420.32 ± 85.72	0.318
Superior (μm)	415.58 ± 91.88	408.23 ± 81.39	0.428
Nasal (μm)	415.45 ± 88.01	410.29 ± 73.26	0.558
Inferior (μm)	416.69 ± 95.67	405.56 ± 85.12	0.250

*Note:* iERM = idiopathic epiretinal membrane; sERM-PRH = secondary epiretinal membrane due to peripheral retinal hole. Temporal, superior, nasal, and inferior = four quadrants of parafoveal area. Independent *t* test.

Abbreviation: CMT, central macular thickness.

**Table 3:** Comparison of opposite quadrants of parafoveal thickness between two groups.

	Temporal (μm)	Nasal (μm)	*p* value
iERM (*N* = 520)	430.03 ± 96.10	415.45 ± 88.01	< 0.001
sERM-PRH (*N* = 115)	420.32 ± 85.72	410.29 ± 73.26	0.214

*Note:* iERM = idiopathic epiretinal membrane; sERM-PRH = secondary epiretinal membrane due to peripheral retinal hole; temporal, nasal = temporal and nasal quadrants of parafoveal area. Paired samples test was done between temporal and nasal quadrants of parafoveal thickness for each eye.

**Table 4:** Comparison of anatomical features based on OCT images between two groups.

	iERM (*N* = 520)	sERM-PRH (*N* = 115)	*p* value
IS/OS junction defect	304 (58.5%)	71 (61.7%)	0.518^b^
Cotton ball sign	75 (14.4%)	20 (17.4%)	0.419^b^
Intraretinal fluid	213 (41.0%)	43 (37.4%)	0.480^b^
EP	15 (2.9%)	4 (3.5%)	0.972^a^

*Clinical severity grading*			
Stage 1	39 (7.5%)	11 (9.6%)	0.193^b^
Stage 2	58 (11.2%)	18 (15.7%)	
Stage 3	250 (48.1%)	51 (44.3%)	
Stage 4	81 (15.6%)	11 (9.6%)	

*Anatomical grading associated with macular holes*			
MPH	15 (2.9%)	5 (4.3%)	0.591^b^
LMH	44 (8.5%)	13 (11.3%)	
ERMF	33 (6.3%)	6 (5.2%)	

*Note:* iERM = idiopathic epiretinal membrane; sERM-PRH = secondary epiretinal membrane due to peripheral retinal hole; IS/OS = the photoreceptor inner/outer segment, ERMF = epiretinal membrane foveoschisis; MPH = macular pseudohole.

Abbreviations: EP, epiretinal proliferation; LMH, lamellar macular hole.

^a^Continuity Correction Test.

^b^Pearson Chi-square test.

## Data Availability

The data used to support the findings of this study are not available due to patient privacy concerns.
